# Multimodal Communication Outcomes for Hispanic Autistic Preschoolers Following Coached Student Clinician and Caregiver-Led NDBIs

**DOI:** 10.3390/bs15101425

**Published:** 2025-10-20

**Authors:** Cindy Gevarter, Jaime Branaman, Jessica Nico, Erin Gallegos, Richelle McGuire

**Affiliations:** Department of Speech and Hearing Sciences, University of New Mexico, Albuquerque, NM 87106, USA; jbranaman@unm.edu (J.B.); jnico@unm.edu (J.N.);

**Keywords:** autism, naturalistic developmental behavioral intervention (NDBI), multimodal, cascading coaching, culturally adapted, neurodiversity affirming

## Abstract

This study examined child outcomes for five minimally verbal (or non-speaking) autistic preschoolers who participated in cascading coaching programs in which naturalistic developmental behavioral intervention (NDBI) techniques were taught to graduate student clinicians and Hispanic caregivers (three who primarily spoke English, and two who spoke Spanish). While prior studies reported on adult participant outcomes, this study analyzed child multimodal communication outcomes, using multiple baselines/probes single case experimental designs across contexts. Neurodiversity-affirming and culturally responsive principles were embedded within the intervention procedures. Following the introduction of a coached NDBI, all five children (three who received the intervention in English and two who received the intervention in Spanish) demonstrated increased use of (a) the total targeted communicative responses and (b) the targeted unprompted communicative responses, across both student clinician-led and caregiver-led play sessions. The Tau-U effect size measures revealed large-to-very large effects across all of the variables. Overall, higher rates of communication responses were observed during student clinician-led sessions than in caregiver-led sessions. Additionally, behavioral coding of the multimodal response forms (e.g., gestures, aided augmentative and alternative communication, signs, vocal words) using the Communication Matrix revealed that the children used a variety of response topographies during the intervention sessions beyond their preferred communication mode (e.g., signs for three participants). Four of the five children used symbolic communication forms consistently across both caregiver and student clinician-led sessions. Importantly, adults’ reinforcement of pre-symbolic or less advanced communication forms during the intervention did not inhibit the use of more advanced forms.

## 1. Introduction

As the prevalence of autism spectrum disorder (ASD) continues to increase, ongoing examination and refinement of evidence-based practices (EBPs) for early communication interventions are necessary. Given a shift towards social models of disability and neurodiversity-affirming approaches, there is a particular need to examine how existing EBPs ethically align with the lived experiences and perspectives of autistic^1^ individuals and their family members (M. Wang et al., 2023). The naturalistic developmental behavioral intervention (NDBI) is one such EBP approach that has been described as having the potential to align with neurodiversity-affirming values (Schuck et al., 2022; M. Wang et al., 2023). An NDBI embeds behavioral and developmental teaching strategies and principles (e.g., prompting, arranging the environment to promote communication, natural consequences) within the context of everyday routines, such as play. Multiple meta-analyses (e.g., Sandbank et al., 2020; Uljarević et al., 2022; Z. Wang et al., 2022) have supported the efficacy of NDBIs for increasing child outcomes in areas such as communication/language, cognition, and adaptive behavior (J. Song et al., 2025). Despite the promise of NDBIs as an EBP that can align with strengths-based, person-centered, and neurodiversity-affirming principles, the aggregated evidence of efficacy for child outcomes does not indicate whether the NDBI implementations described in the literature have incorporated these values (Schuck et al., 2022; M. Wang et al., 2023). For instance, there are concerns that if not adapted appropriately, NDBI research can: (a) fail to meet the needs of culturally and linguistically diverse (CLD) families, (b) lack documentation of individual and family involvement in the planning and implementation of the NDBI, and (c) focus primarily on neurotypical goals (Schuck et al., 2022; M. Wang et al., 2023).

Importantly, although recent scoping reviews highlight increased exploration of culturally adapted caregiver-led interventions and NDBIs, they also suggest that additional research is critical to meet the needs of a diverse range of autistic children and their families (Douglas et al., 2024; DuBay, 2022). This includes, for example, adapting NDBIs to align with the cultural values of both English and Spanish-speaking Hispanic/Latino^2^ families. In addition, cultural individualization of NDBIs requires family involvement (Douglas et al., 2024; M. Wang et al., 2023). Unfortunately, J. Song et al. (2025) indicated that about 45% of studies across NDBI meta-analyses do not involve a caregiver in the implementation process. Regarding a focus on neurotypical goals, one important variable to consider is whether NDBI research places greater value on the teaching of natural speech in comparison to multimodal forms, such as augmentative and alternative communication, or AAC (Gaddy & Crow, 2023; M. Wang et al., 2023). Disability advocacy groups and autistic individuals have emphasized the importance of teaching, honoring, and providing access to multiple forms of communication, including AAC (Donaldson et al., 2017; National Joint Committee for the Communication Needs of Persons with Severe Disabilities, 2024). Formal AAC systems can include unaided/topography-based approaches (e.g., sign language), as well as a variety of non-electronic and electronic-aided/selection-based systems (e.g., speech-generating devices or SGDs). Although not typically considered formal AAC, but rather intentional pre-symbolic communication forms, unaided/topography-based forms can also include unconventional forms (e.g., reaching, leading, using non-word vocalizations) and conventional gestures, such as pointing (Rowland, 2011).

As unaided and aided systems have differential advantages and disadvantages across different contexts and communication purposes, teaching multimodal communication offers many benefits (Beukelman & Light, 2020). Teaching and responding to multiple modalities are particularly critical for autistic children described in the literature as non-verbal^3^ or minimally verbal (Koegel et al., 2020). Although varying definitions of these terms exist, most definitions focus on children using between 0–50 vocal speech words in functional contexts (Koegel et al., 2020). Amongst early intervention and school-age populations, approximately 25–35% of children with ASD use between 0–30 natural speech words (Kasari et al., 2013; Rose et al., 2016). Additional research suggests that around 57% of autistic school-age children are rated by special educators as either non-proficient in their use of vocal speech (34%) or are described as primarily relying upon other forms of communication (23%; Andzik et al., 2018). In this same study, 96% of children described as primarily relying on gestures to communicate were also rated as being non-proficient with this modality (Andzik et al., 2018).

Although it has been suggested that NDBI research demonstrates fewer clear benefits for minimally verbal or non-speaking autistic children, synthesized results across multiple meta-analyses indicate that a significant relationship does not exist between pre-study vocal language skills and post-intervention language and communication outcomes (J. Song et al., 2025). However, it is important to note that studies have differentially defined language and communication outcomes (e.g., some focus only on natural speech outcomes, while others include AAC or gestures). Systematic reviews examining the effects of naturalistic interventions on prelinguistic communication (Dubin & Lieberman-Betz, 2020) and aided AAC (Gevarter & Zamora, 2018; Pope et al., 2024) suggest that an NDBI is effective for increasing communication outcomes beyond vocal speech. However, two reviews (Dubin & Lieberman-Betz, 2020; Gevarter & Zamora, 2018) note a paucity of research involving caregivers, and Pope et al. (2024) excluded caregiver-implemented NDBIs.

NBDIs often make use of caregiver coaching in which clinicians embed the teaching of instructional techniques within the context of selected natural routines. Caregiver coaching also involves elements such as joint planning, modeling, and feedback (Gevarter et al., 2021; Gallegos et al., 2024). Cascading coaching models, in which a highly trained researcher or clinician first coaches a less experienced clinician (e.g., graduate student) who then provides coaching to caregivers, have also been explored in NDBI literature (e.g., Gallegos et al., 2024; Gevarter et al., 2021; McGuire et al., 2025; Meadan et al., 2020).

Despite the importance of teaching caregivers to promote communication outcomes during NDBIs, a recent review of 17 caregiver-mediated single-case experimental design NDBI studies (including coaching models) indicated that only 10 studies specifically examined outcomes related to language or communication (van Noorden et al., 2024). Upon a review of these ten studies, the current study’s authors found that although nine of the ten studies included at least one participant that could be described as minimally verbal or non-speaking, only three studies included response forms other than natural speech in the child communication outcomes (Cain, 2017; Gevarter et al., 2021; Ousley, 2022). Similarly, in a review of randomized control trials of parent-mediated NDBIs, most studies used outcome measurements involving formal language tools that typically do not assess non-speech forms (Y. Song et al., 2025).

Although existing reviews related to cultural adaptations in NDBI and caregiver-mediated interventions do not explore the intersectionality between culture and child communication modalities, they do emphasize the importance of involving caregivers in all aspects of the intervention (e.g., modality selection) and utilizing culturally relevant assessment tools to measure outcomes (Douglas et al., 2024; DuBay, 2022). Unfortunately, several existing parent-mediated NDBI studies involving Hispanic families have either not included data related to child outcomes (e.g., Gallegos et al., 2024; McGuire et al., 2025; Meadan et al., 2020), have combined child outcomes with adult outcomes (Gevarter et al., 2021), or have reported null effects on child communication (Lopez, 2024). Pickard et al. (2024) did report significant increases in parent-reported child social communication skills, using a measure that included multiple communication forms (Social Communication Checklist; SCC; Ingersoll & Dvortcsak, 2019), but there were no (a) reported independently observable child communication outcomes, (b) data on children’s total use of communication versus unprompted communication, or (c) specific information regarding which communication modalities children used.

Despite growing interest in culturally adapted NDBIs, there is a critical gap in the research examining child-level communication outcomes, particularly for bilingual or non-English speaking children. In addition to examining the effects of NDBIs on children’s use of prompted and unprompted communicative responses, methods for reporting variability in communication modalities are needed. One culturally adaptable tool that can be used to describe multimodal communication forms is the Communication Matrix (Rowland, 2011). The Communication Matrix uses communication levels to describe differences in the topography, intentionality, symbolism, pragmatic function, and syntactical complexity of communicative responses. Levels III–VI include intentional communication forms that are described as pre-symbolic (e.g., unconventional and conventional gestures; levels III and IV, respectively), concrete symbols (e.g., aided AAC; level V), and abstract symbols (e.g., signs, vocal words; level VI) that do not yet involve the building of sentences. Level III has more limited pragmatic functions (e.g., focused primarily on early demands/requests) and level IV also excludes tacting forms (e.g., naming objects and commenting). The Communication Matrix can be used either as a parent reporting tool or as a behavioral observation tool (e.g., coding responses according to the communication level). The culturally adaptivity of the Communication Matrix is enhanced by the fact that it has been translated into multiple languages (including Spanish), allows caregivers to describe a variety of communication forms that may be culturally bound, and is freely available online for parents and caregivers.

This study aimed to examine the effects of coached NDBIs on multimodal communication outcomes for minimally verbal Hispanic autistic preschoolers, using behavioral observations and Communication Matrix coding across student clinician- and caregiver-led sessions. Specifically, researchers analyzed child data from two prior studies involving cascading NDBI coaching given to graduate students, Hispanic caregivers, and Hispanic autistic preschoolers (Gallegos et al., 2024; McGuire et al., 2025). Both prior studies demonstrated functional relationships between cascading coaching and adult participants’ increased use of NDBI techniques. Gallegos et al. (2024) included English-speaking caregivers, while McGuire et al. (2025) involved Spanish-speaking caregivers and bilingual graduate student clinicians. Although the prior studies included social validity measures (e.g., surveys or qualitative interviews) that indicated that both student clinicians and caregivers reported positive changes to the children’s communication skills, observational data on the child outcomes were not included. Notably, this study is novel in its use of cascading coaching involving bilingual student clinicians and caregivers, its inclusion of both English- and Spanish-speaking families, its application of Communication Matrix coding to track multimodal outcomes, and its integration of neurodiversity-affirming and culturally responsive practices. To examine the child communication outcomes of this novel approach, the researchers aimed to answer the following research questions:

1. What were the effects of cascading coaching programs involving student clinician-led and caregiver-led NDBIs on Hispanic autistic preschoolers’ use of the total targeted communicative responses (prompted and unprompted) and unprompted targeted communicative responses?
2. Did Hispanic autistic preschoolers use a variety of communicative topographies (across different Communication Matrix levels) in student clinician- and caregiver-led NDBI contexts?

## 2. Materials and Methods

### 2.1. Participants

The participants included five minimally verbal or non-speaking autistic Hispanic preschoolers who had participated in prior cascading coaching studies (Gallegos et al., 2024; McGuire et al., 2025). The children were recruited via outreach to local early intervention agencies, preschools, private speech–language pathologists (SLPs), and parent Facebook groups, during the prior studies.

At the time of each initial study, participants met the following criterion: (a) aged 2.5–4.11 years old, (b) had an independent diagnosis of ASD or was on a waiting list for diagnosis but showed signs of ASD confirmed via the Childhood Autism Rating Scale second edition (CARS-2; Schopler et al., 2010) or the Spanish version of the Gilliam Autism Rating Scale third edition (GARS-3; Gilliam, 2014), (c) was reported to use fewer than 30 spoken words, and (d) had a caregiver who was willing to participate in coaching. The three participants in study 1 (Gallegos et al., 2024) were required to live in homes where English was the primary language spoken, and the two participants in study 2 (McGuire et al., 2025) were required to live in a home where Spanish was the primary language spoken.

Table 1 outlines the demographic and assessment information for the child participants (with the same pseudonyms assigned in prior studies).

#### Screening Assessments

During the screenings for the initial studies, researchers interviewed the parents of the child participants. For 4 of the 5 participants, the English version of the CARS-2 (which has demonstrated reliability and validity; Reszka et al., 2014) was administered in order for the parent to describe their child’s ASD characteristics. Zandra’s mother completed the Spanish version of the GARS-3 (which has demonstrated reliability and validity across the different measures; Schmidt et al., 2023). The parents additionally provided information about the participant’s communication skills via the English or Spanish version of the Communication Matrix (Rowland, 2011), and the Vineland Adaptive Behavior Scale 3rd edition (VABS-III; Sparrow et al., 2016). Parents of 4 of the 5 participants responded to the communication sections of the VABS-III interview form in English, and Zandra’s mother used the Spanish version of the caregiver’s form of the VABS-III. Finally, the researchers used English and Spanish versions of the Reinforcer Assessment for Individuals with Severe Disabilities (RAISD; Fisher et al., 1996, 2021) to determine the preferred items for the play-based intervention.

### 2.2. Researchers and Interventionists

In the prior studies, a lead instructor (BCBA-D, an associate professor in a speech and hearing sciences [SHS] department and the first author) provided instructions and coaching to the student clinicians working with the child participants and caregivers. Authors four and five (lead authors of the prior studies, which were completed to meet master’s thesis requirements) coded the primary dependent variables in this study. Authors two and three (PhD students specializing in autism in the SHS department) completed the data analysis, alongside the first author. Additional graduate and undergraduate students served as interobserver agreement coders for the study outcomes.

The primary interventionists in this study included: (a) graduate-level student clinicians studying either SHS (three students) or special education (two students), and (b) child caregivers (four mothers and one grandmother). The caregivers in study 1 all primarily spoke English. Although one of the student clinicians in study 1 was bilingual (spoke Spanish and English), she did not apply her Spanish skills in the context of the study. In study 2, the student clinicians were both bilingual English/Spanish speakers and the caregivers were monolingual Spanish speakers. Further demographic information about the student clinicians and caregivers can be found in the prior studies (Gallegos et al., 2024; McGuire et al., 2025).

### 2.3. Setting and Materials

The research sessions took place in child-friendly university clinic rooms that each contained a carpeted play area, table and chairs, and additional research materials/preferred items further described below. Caregivers received USD 10 Amazon gift cards for each research session they attended with the child participant. Visual supports included English and Spanish versions of (a) instructional visual aids that outlined NDBI techniques, (b) activity planners used to collaboratively plan sessions jointly with clinicians and families, and (c) coaching checklists (see Gallegos et al., 2024; McGuire et al., 2025). Child-specific sets of play materials (one set for student clinician-led sessions, and another for caregiver-led sessions) were created using parent-provided information from the RAISD (Fisher et al., 1996, 2021). Small, preferred items (e.g., toy cars, playdough, water toys, books, puzzles) were placed in the clinic rooms, inside a bag, at the beginning of each session, while larger preferred activity items (e.g., water table, trampoline) remained in the research room throughout the sessions. As part of a cultural adaptation for Zandra, food items (e.g., gummies, pizza) were added to the caregiver-led sessions (see McGuire et al., 2025). Zandra was also the only participant for whom an aided AAC system (an iPad featuring the Proloquo2go AAC application by Assistiveware) was added during the coaching phase.

Materials for the data analysis and coding included: (a) video recordings of the baseline and coaching sessions from the prior studies, (b) initial data sheets developed for the prior studies that included time stamps for observed targeted communication temptations used by adults, and (c) additional data sheets developed specifically for this study.

### 2.4. Research Design

The two prior studies utilized a series of multiple baseline and multiple probe across (adult) participants single case experimental designs (Gast et al., 2014). The child outcome data described in this study would best be described as a series of multiple baselines and multiple probes designs across contexts (i.e., student clinician-led play sessions and caregiver-led play sessions). It is important to note, however, that decisions regarding when to begin an intervention in caregiver-led contexts were based on increases in the student clinicians’ use of NDBI techniques (and the time constraints of the study) rather than child outcome measures.

Initial research sessions took place across two iterations of a four-week grant-funded summer clinic for young autistic children. Three days a week, the participants in this study and their student clinicians participated in group clinic sessions (which included additional non-participating children) that included common preschool activities and routines. Prior to the start of daily group sessions, the children participated in caregiver-led play sessions (with student clinicians present). At various points throughout the group session, children transitioned from the group to participate in individual student-led play sessions (with the lead instructor present until the last week of sessions) at least one hour after the caregiver-led sessions. Baseline data collection (with probes used in study 2) occurred across both student and caregiver-led play contexts during week 1. In week 2, child baseline data continued to be collected in caregiver-led contexts, while intervention data collection began in student clinician-led contexts (i.e., after student clinicians had begun receiving coaching and instruction on NDBI techniques from the lead instructor). During weeks 3 and 4, intervention-related child outcomes were collected across both student and caregiver-led contexts (i.e., after the student clinicians had begun coaching the caregivers to use NDBI techniques during caregiver-led sessions). For participants who completed all of the research sessions, the lead instructor was no longer present during week 4 of the student-led sessions, but the student clinicians continued to be present/coach parents during week four caregiver-led sessions. Illness-related absences led to some participants taking part in fewer than the 12 expected sessions (including baseline sessions and the intervention) with both their assigned student clinician and their caregiver.

### 2.5. Dependent Variables

The primary dependent variables were the child’s (a) rate per minute of total targeted communicative responses (prompted and unprompted) to adult participant targeted communication temptations and (b) the child’s rate per minute of targeted unprompted communicative responses to targeted adult participant communication temptations. Secondary variables examined the rate of total communication responses across a given Communication Matrix level during the baseline and intervention sessions (for both student clinician-led and caregiver-led sessions). The Communication Matrix levels coded included: level III responses (unconventional communication), level IV responses (conventional gestures), level V responses (concrete symbols), and level VI responses (abstract words or signs). Level VI responses were further coded as either a vocal word or sign.

### 2.6. Data Collection

#### Coding and Interobserver Agreement (IOA)

Using video recordings and two standardized data collection sheets, coders documented the components of dependent variables. The frequency of targeted prompted and unprompted communicative responses was first recorded on a data sheet that was used to collect data on adult behaviors in the two prior studies. Using operational definitions for prompted and unprompted communicative responses, coders first indicated whether the child’s last communicative act in response to a targeted temptation was prompted or unprompted. Prompted responses were coded when the communicative act involved a model, gestural, or physical cue, and unprompted responses excluded these cues. Student clinicians and parents were not taught to use verbal prompts or directives. Cues such as “tell me what you need” were not considered prompts, but rather natural cues. To determine the total number of targeted communicative responses per session, coders added the total number of prompted and unprompted communicative responses that occurred after adult target temptations occurred.

To code for the Communication Matrix level of a response, researchers created secondary data sheets with time stamps indicating when targeted communication temptations were coded in the prior study. Using knowledge from the prior studies (i.e., possible communication responses student clinicians and caregivers had listed during joint planning sessions, prior viewing of videos) researchers created individualized descriptions of the topographies each child participant used (e.g., conventional gestures, signs) and categorized these responses according to their level on the Communication Matrix. These lists were then used to create operational definitions of level III–VI responses that included individualized examples for each child. Coders used this information to indicate the Communication Matrix level (breaking up level VI into signs vs. vocal words) for each targeted child communicative act on the data sheets. For each research session, coders totaled the number of targeted communicative responses at each Communication Matrix level (e.g., 4 targeted level III responses, 10 targeted level V responses).

Researchers trained secondary coders to conduct interobserver agreement (IOA) checks by reviewing all of the operational definitions, practicing coding videos not selected for an IOA in a group context, and independently coding additional practice videos (1–2 videos) to meet an 80% agreement criterion with the original coders. Next, for each child participant, researchers randomly selected 33% of videos (across phases and adult interventionists) not used for coding practice for the IOA checks. The number of agreements between coders was divided by the total amount of disagreements + agreements and multiplied by 100. The average IOA scores for targeted prompted and unprompted responses were as follows: 95.67% (Garrett); 94.97% (Michelle); 94.27% (Vince); 94.33% (Gustavo); and 89.82% (Zandra). The average IOA scores for the Communication Matrix levels were as follows: 97.67% (Garrett); 94.97% (Michelle); 90.65% (Vince); 95.80% (Gustavo); and 91.24% (Zandra).

### 2.7. Procedures

#### 2.7.1. Baseline and Target Selection

In both prior studies, adult participants were instructed to interact/play with the child as they normally would during the baseline sessions. The lead instructor was present during student clinician-led sessions, but did not provide any instructions or feedback. Similarly, student clinicians were present during caregiver-led sessions, but did not provide instructions or feedback. Play observations (which began as soon as the child showed interest in an item or an activity) occurred for 12 min in study 1 (Gallegos et al., 2024) and for 15 min in study 2 (McGuire et al., 2025). The session length was increased in the second study based upon student clinician feedback from the prior study. The only other difference between the baseline sessions in the two original studies is that the sessions were conducted in English in study 1 and in Spanish in study 2. In both studies, researchers reviewed the baseline sessions and selected target communication temptations for each adult participant that (a) occurred at a low frequency during the baseline session and (b) were appropriate for the child’s interests/activities. Researchers then used the baseline observations and pre-baseline Communication Matrix reports to brainstorm lists of multimodal communication forms each child might use in response to selected temptations. These communicative forms were further discussed and refined during the joint planning component of coaching sessions (see Intervention). It should be noted that due to the short length of the study, the intention was not to introduce new communication forms, but rather to expand upon the use, function, and vocabulary of existing topographies. The only exception to this was Zandra, for whom aided AAC was introduced to align with caregiver and client preferences (e.g., the child showed a physical aversion to unaided prompting forms, and her mother wanted her to expand beyond her level III responses). Table 2 outlines the targeted temptations used, along with example child responses.

#### 2.7.2. Intervention

For more detailed descriptions of the NDBI cascading model (i.e., researcher provided NDBI instructions and coaching for student clinicians and student clinician coaching of caregivers), please see the prior studies (Gallegos et al., 2024; McGuire et al., 2025). Procedural integrity data is reported in these studies and McGuire et al. also provide a more detailed summary of the cultural adaptations used to meet the needs of Spanish-speaking caregivers. Below we provide a summary of the procedures used across both studies, while noting any differences between the studies.

In both prior studies, at the start of week two, the lead instructor provided students with NDBI instructions during a university course, using a behavioral skills training (BST) model (Miltenberger, 2011). Instructional components included verbal descriptions of NDBI techniques enhanced by visual supports (activity planners, instructional aids), in situ models/video models, and small group role play and feedback. The only differences between the instructions given in study 1 (Gallegos et al., 2024) and study 2 (McGuire et al., 2025) was that the second study included: (a) Spanish visual supports, (b) opportunities to select Spanish terminology with their peers, and (c) additional instructions focused on allowing children time to play/interact with materials before moving to creating another temptation.

After the group instructions were given, the lead instructor began coaching students during their sessions with the child participants. Coaching sessions lasted approximately 30 min, including the 12–15 min play observations. In both studies, the NDBI elicitation techniques taught included (a) using a targeted temptation, (b) waiting at least 3 s prior to prompting the child to communicate, and (c) using a prompt when the child did not initiate communication on their own. Response techniques used in both studies also included (a) naturally reinforcing the child’s communicative responses and (b) pairing reinforcement with a vocal speech model mapped onto the communicative act. In study 1, the vocal speech models needed to be grammatically complete (e.g., *You want to jump*), but due to caregiver challenges associated with learning to use this technique in such a short time, this requirement was removed in study 2. Additionally, study 2 also explicitly focused on teaching adult participants to allow children time to engage/interact with materials prior to introducing a new temptation as an additional response technique.

Across both prior studies, the first three coaching/intervention sessions involved the lead instructor (a) introducing and modeling one new targeted temptation; (b) using an activity planner to jointly discuss and determine the activities, child responses, prompt options, and natural speech models that were appropriate for the selected temptation; (c) verbally describing and modeling the NDBI elicitation and response techniques; (d) providing, as needed, assistance (e.g., with materials, prompting, redirection), verbal cues, and feedback during the play observation; and (e) offering constructive/positive feedback and time for questions after the play observation. Study 2 included Spanish visual supports and time to plan, model, and practice ways to ensure the children had adequate time to interact with the materials prior to introducing another temptation.

In both studies, student clinician and caregiver input was obtained during the joint planning, which led to the selection of potential prompt types and response forms across a variety of communication forms and modalities. For example, acceptable response forms for Garrett (study 1) included level III and level IV gestures (reach, point) and single words (*go*), while acceptable response forms for Zandra (study 2) included gestures (guiding hand), persistent vocalizations, aided AAC responses, and single words (*agua*). Researchers did not require adult participants to follow a specific, structured prompting hierarchy, in part due to the short duration of the cascading coaching intervention and the focus on maintaining naturalistic play. Rather, adult participants were provided with explicit instructions and real-time feedback on ways to provide responsive feedback to the child to meet and grow their communication skills. For instance, during the group instruction and coaching session, the lead instructor provided student clinicians with examples, models, and rationales for when they may want to use a less restrictive or a more restrictive prompt. Additionally, within the play sessions, the lead instructor provided real-time cueing to student clinicians regarding when they should: (a) accept a communicative act corresponding to a lower level on the matrix (e.g., level III–IV), (b) try prompting a higher-level form, (c) wait longer than 3 s for a response, or (d) try a different prompt. For example, if a child showed signs of aversion or frustration with a prompt to use an AAC device or sign during a previous temptation, the lead instructor may have suggested accepting a gesture for the next temptation or adjusting the prompt type. Conversely, if a child responded to several signs or aided AAC model prompts in a row, the coach might suggest increasing the wait time for the next temptation.

### 2.8. Data Analysis

The rate per minute of total targeted communicative responses (prompted and unprompted), as well as unprompted communicative responses, were plotted across the baseline and intervention (coaching) phases. Visual analysis methods described by Kratochwill et al. (2013) were used to ascertain differences in the immediacy of the effects, levels, variability, and trends between the baseline and intervention phases. To complement the visual analysis, researchers used a web software package to calculate the Tau-U, a non-overlap effect size measure (Vannest et al., 2016). In addition to considering changes in the levels and trends between the phases, the Tau-U measure enables corrections for positive baseline trends (Brossart et al., 2014; Rakap, 2015). Corrections were used for the following participants and variables: Vince with clinician, Michelle with clinician, and Gustavo with caregiver. The effect sizes are categorized as small (scores of 0.20 and below), moderate (0.20–0.60), large (0.60–0.80), and very large (0.80 and above) (Vannest & Ninci, 2015).

To analyze participants’ multimodal communicative topographies across the different phases, researchers calculated the rate (per session) of targeted communicative responses corresponding to each coded Communication Matrix level for all the baseline and intervention sessions for each participant and context (e.g., student clinician-led sessions, caregiver-led sessions). Inferential statistics were considered for the analysis, but were ruled out due to violating parametric assumptions (e.g., normality, homogeneity of variance) and reduced reliability of the nonparametric measures resulting from the small dependent sample size. Instead, descriptive statistics (i.e., average rate of responding at each level during the baseline and intervention) and stacked bar graphs were used to analyze and visualize communicative response form use across the different phases, contexts, and participants.

## 3. Results

### 3.1. Targeted Child Communicative Responses

The findings across all five participants demonstrated functional relationships between the NDBI cascading coaching program and both the children’s rate of total targeted communicative responses per minute and the rate of unprompted targeted communicative responses per minute. Although there was variability in terms of the levels of response and the presence of trends within and across participants, all but two Tau-U effect size measures indicated very large effects. A large effect was observed for Gustavo’s use of unprompted communicative responses during sessions led by both his student clinician and grandmother. With only some exceptions across different participants and contexts, the levels of unprompted responding tended to only be slightly below the total levels of responding. In some cases, there was also less differentiation between the two variables (and some overlap) in later intervention sessions, indicating relative increases in the proportion of unprompted responses over time. Additionally, the child participants tended to have higher overall rates of responding to student clinicians as compared to caregivers. This finding corresponds to student clinicians’ overall higher rates of NDBI elicitation and response technique use as compared to caregivers that was reported in the prior related studies (Gallegos et al., 2024; McGuire et al., 2025). The detailed results are described for each participant below.

#### 3.1.1. Garret’s Targeted Communicative Response Results

**Figure 1:**
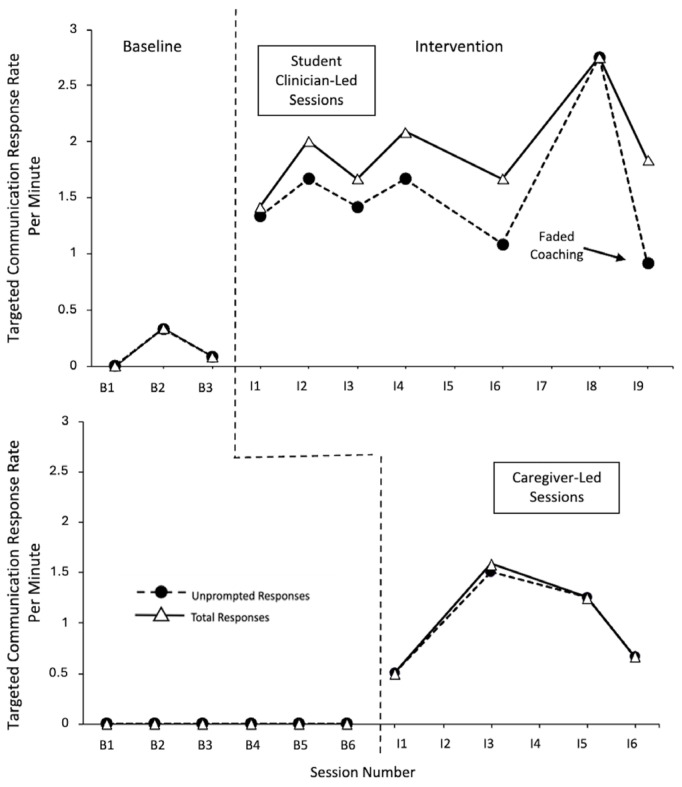
Garrett’s rate of unprompted and total targeted communication responses per minute across 12 min student clinician-led sessions and caregiver-led sessions.

The data for Garrett is displayed in Figure 1. A very large effect size of 1 was seen for unprompted and total responses with the student clinician (CI = 0.313–1), along with unprompted and total responses with his caregiver (CI = 0.357–1).

Garrett demonstrated low, stable levels of responding (unprompted and total) within the baseline probes across the student clinician-led and caregiver-led sessions. Within the intervention phases, there were strong demonstrations of the immediacy of the effect, with a large increase in the response level upon initiation of the intervention phase within the student clinician-led sessions, and a moderate increase in the response level within the caregiver-led intervention sessions. Within the student clinician-led intervention sessions, Garrett demonstrated high rates of unprompted and total responses, with minimal-to-moderate variability, and an overall positive trend. For the majority of the sessions, the level of unprompted responses per minute was slightly below the level of total responses per minute (with the exception of intervention session 7 when all the responses were independent and session 8 when there was a decrease in unprompted responding). Within the caregiver-led intervention sessions, Garrett demonstrated moderate levels of unprompted and total responses, with moderate-to-high variability, and no trend. It should be noted that, while the responses remained above the baseline levels, decreases in the unprompted and total response rates were observed during intervention session 8, in view of the child’s observed satiation with the intervention materials, and faded coaching. Garrett primarily used unprompted responses with his caregiver (i.e., an overlap between unprompted and total responding).

#### 3.1.2. Vince’s Targeted Communicative Response Results

**Figure 2:**
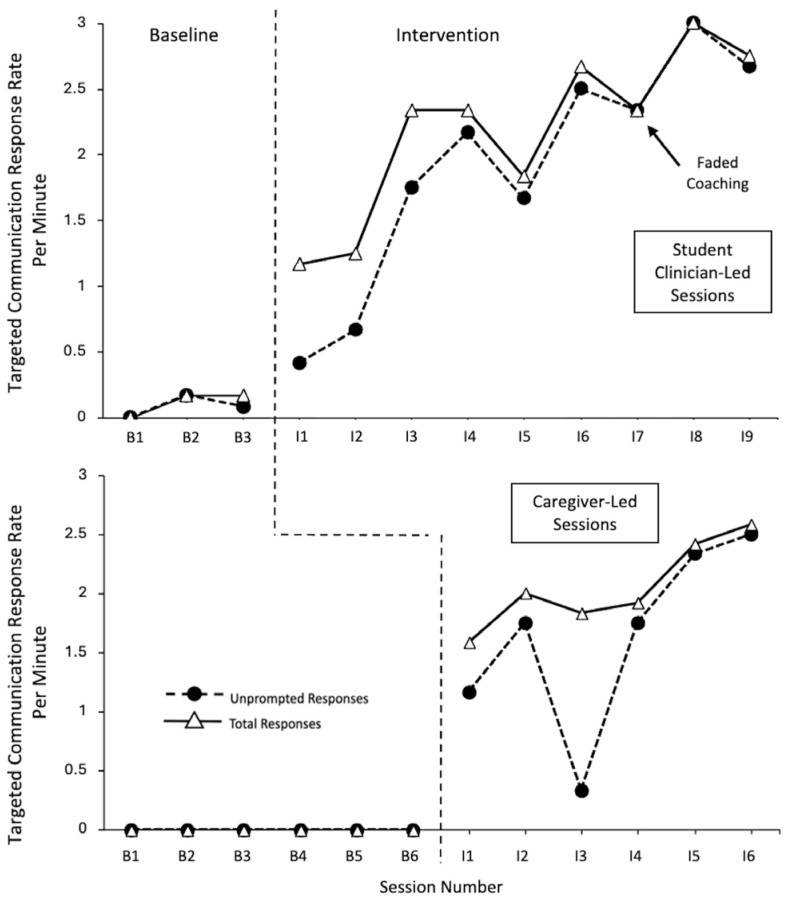
Vince’s rate of unprompted and total targeted communication responses per minute across 12 min student clinician-led sessions and caregiver-led sessions.

Figure 2 displays the data for Vince. His data demonstrate very large effect sizes across different contexts. Using a correction for the baseline trend in regard to the conditions with his student clinician, Vince had an effect size of 0.963 for the rate of unprompted responses with the student clinician (CI = 0.305–1), 0.9259 for the rate of total responses with the student clinician (CI = 0.267–1), and 1 for both the unprompted and total response rates with his caregiver (CI = 0.429–1).

Vince demonstrated low, stable levels of response per minute (unprompted and total) within the baseline probes across the student clinician-led and caregiver-led sessions. Within the intervention phases, an immediacy of the effect can be seen with a large increase in the response level across student clinician- and caregiver-led contexts for the total responses per minute and for unprompted responses per minute in the caregiver-led sessions. The immediacy of the effect was more gradual for unprompted responses during the first two student clinician-led sessions. Within the student clinician-led intervention sessions, Vince demonstrated continuously increasing levels of unprompted and total responses rates, with minimal variability, and a strong positive trend. Over time, there was less differentiation between the levels of unprompted and total responding, indicating a shift towards more unprompted responding. Within the caregiver-led intervention sessions, Vince demonstrated high rates of unprompted and total responses, with low variability, and a steady positive trend. It should be noted that a decrease in unprompted responses can be seen within caregiver-led intervention session 3, as the child required increased support and more prompting due to a new temptation being introduced, and a new corresponding sign being targeted during that session. Otherwise, over time, there was less differentiation between the levels of unprompted and total responding rates, indicating a shift towards more unprompted responding.

#### 3.1.3. Michelle’s Targeted Communicative Response Results

**Figure 3:**
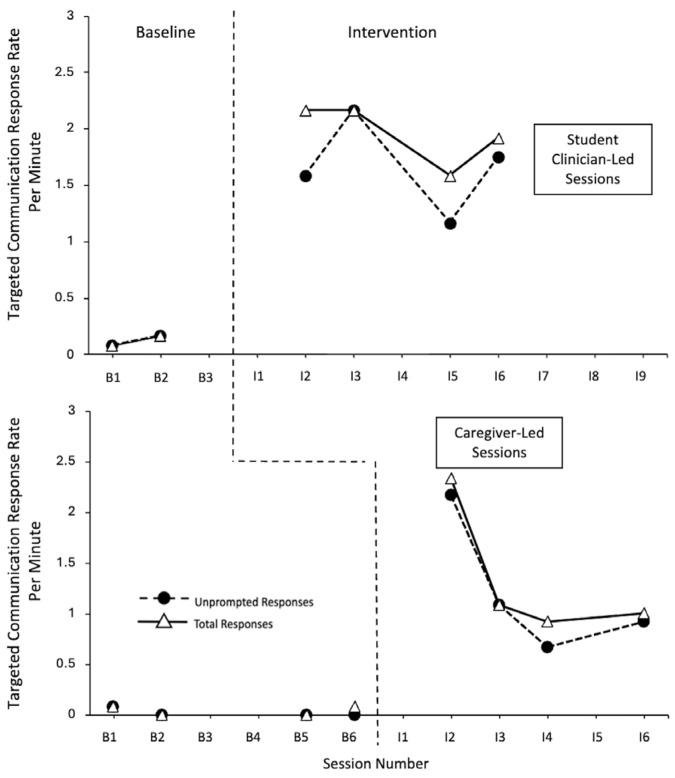
Michelle’s rate of unprompted and total targeted communication responses per minute across 12 min student clinician-led sessions and caregiver-led sessions.

Michelle’s response data is illustrated in Figure 3. Her data indicates very large effect sizes across different contexts. Using the correction for the baseline trend during the student clinician conditions, the effect size was 0.875 for the unprompted and total response rates during the student clinician sessions (CI = −0.013–1), and 1 for the unprompted and total responses with her caregiver (CI = 0.288–1).

It should be noted that Michelle had multiple illness-related absences throughout the baseline and intervention phases. This led to scheduling challenges, which resulted in a lack of faded coaching within the student clinician-led intervention. Michelle demonstrated low levels of unprompted and total responses throughout the baseline phase across both conditions (student clinician led, caregiver led). Within the intervention phases, strong immediate effects can be seen across the student clinician-led and caregiver-led sessions, with high levels of unprompted and total responses. Within the student clinician-led intervention sessions, Michelle demonstrated moderately high levels of unprompted and total response rates, with minimal variability, and an overall neutral trend. Variability in terms of differences in the response levels between the two dependent variables were observed, but the differences were not large, and an overlap was demonstrated. Within the caregiver-led intervention sessions, Michelle initially demonstrated high levels of unprompted and total response rates during her first intervention session, which then decreased. After this initial decrease, low variability and a neutral trend occurred. During this time, researchers observed Michelle showing signs of satiation with some of her play items. The overall differences between the levels of unprompted and total rates of responding were not large and an overlap was demonstrated.

#### 3.1.4. Gustavo’s Targeted Communicative Response Results

**Figure 4:**
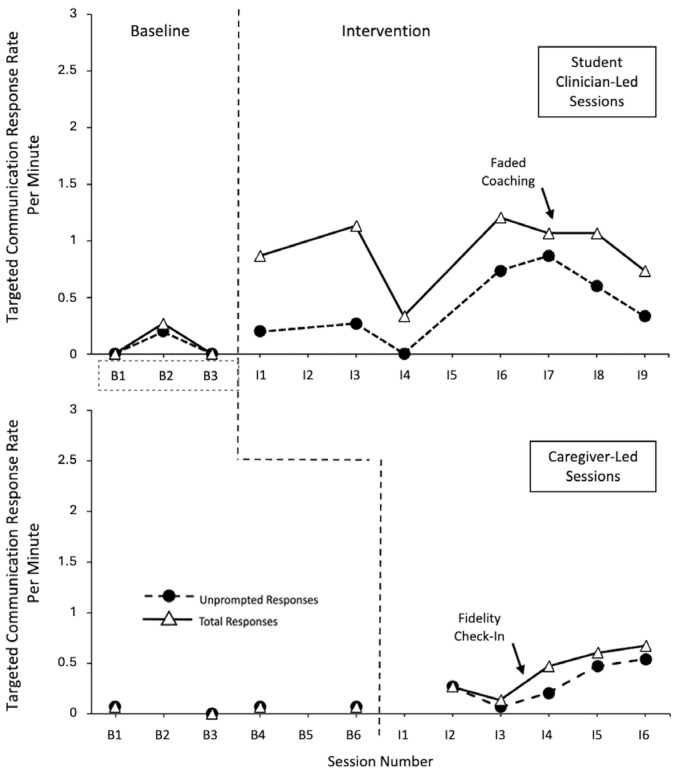
Gustavo’s rate of unprompted and total targeted communication responses per minute across 15 min student clinician-led sessions and caregiver-led sessions.

The data for Gustavo is displayed in Figure 4. His effect sizes varied the most across different contexts. The data showed large effect sizes for the unprompted response rates during the student clinician and caregiver-led sessions. The effect size for responses with the student clinician was 0.7619 (CI = 0.075–1) and 0.8 with his caregiver (CI = 0.128–1). There were very large effect sizes for the total responses per minute with the student clinician with a value of 1 (CI = 0.313–1) and, for the caregiver, with a value of 0.95 (CI = 0.278–1). It is noted that both of the caregiver values had a correction applied in regard to the baseline.

Gustavo demonstrated low, relatively stable levels of responses (unprompted and total) within the baseline probes across the student clinician-led and caregiver-led sessions. For the student clinician-led sessions, although there was an immediacy of the effect for the total response rate, an overlap between the baseline and intervention unprompted response rate data points can be seen during the early student clinician-led intervention sessions. These results were impacted by (a) the fact that new temptations (and corresponding new response forms) were introduced during each of these sessions and (b) intervention session 4 was cut short (9:30 min in duration) due to signs of child illness and the child withdrawing assent. Overall, however, Gustavo demonstrated increasing levels of unprompted and total response rates during the student clinician sessions, with minimal variability outside of the shortened session, and a slight positive trend, prior to a slight decreasing trend during the final sessions. Gustavo’s data indicates clear differentiation between the total response and unprompted response rates; however, this differentiation decreased over time (e.g., proportionately more unprompted responding in later sessions).

Within the caregiver-led sessions, there were more gradual changes in the level of responding, with some overlap between the baseline and intervention. This overlap occurred when his caregiver was provided with instructions on a new communication temptation. Additionally, a fidelity check-in with the student clinician was also conducted after lower levels of responding were observed (see McGuire et al., 2025 for further details). Overall, during the sessions with his grandmother, Gustavo demonstrated a gradual increase in his response rate levels, with minimal variability and positive trends in terms of both his unprompted and total responses. Although Gustavo initially showed less differentiation between his unprompted and total response rates during his first two sessions, this also occurred when his grandmother tended to accept independent level III or VI responses rather than prompt level VI responses. For the remaining sessions, although his total response rate was higher than his unprompted responding, the gaps were not large.

#### 3.1.5. Zandra’s Targeted Communicative Response Results

**Figure 5:**
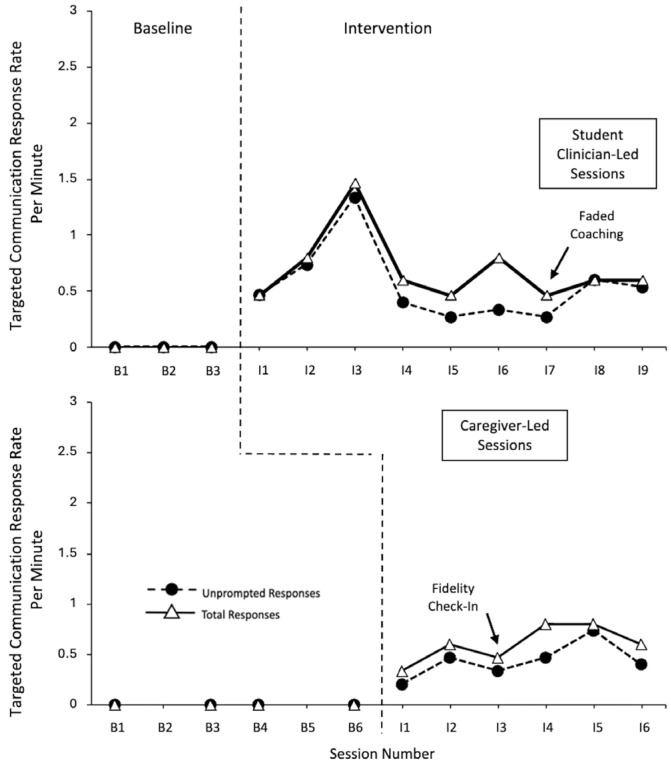
Zandra’s rate of unprompted and total targeted communication responses per minute across 15 min student clinician-led sessions and caregiver-led sessions.

The data for Zandra’s response rates are shown in Figure 5. The data indicates very large effect sizes across different contexts. Both her unprompted and total response rates with the student clinician had a value of 1 (CI = 0.341–1) and a value of 1 (CI = 0.357–1) was recorded with the caregiver. Zandra demonstrated low, stable levels of responding (unprompted and total) within the baseline probes across the student clinician-led and caregiver-led sessions. Within the student clinician-led intervention sessions, Zandra demonstrated immediate changes in her levels of responding that were maintained for both the unprompted and total response rates throughout the intervention phase, with an overall neutral trend. A large increase in the levels of responding can be seen during the third intervention session, due to Zandra demonstrating a particularly strong interest in independently requesting more water toys that day and the student clinician following her lead. Overall, some overlap between the unprompted and total response rates was observed, with some limited differentiation between variables (indicating proportionately high use of unprompted responding).

For the caregiver-led sessions, there was also an immediate change in level for both variables. The first intervention session only includes data from 9 min of the intervention due to a researcher recording error. Overall, the child maintained their level of responding across both variables, with low variability and a slight positive trend, prior to a slight decrease during the last session. There was a slight differentiation between the unprompted and total response rates that were maintained throughout most of the sessions.

### 3.2. Communication Matrix Level Response Rates

**Figure 6:**
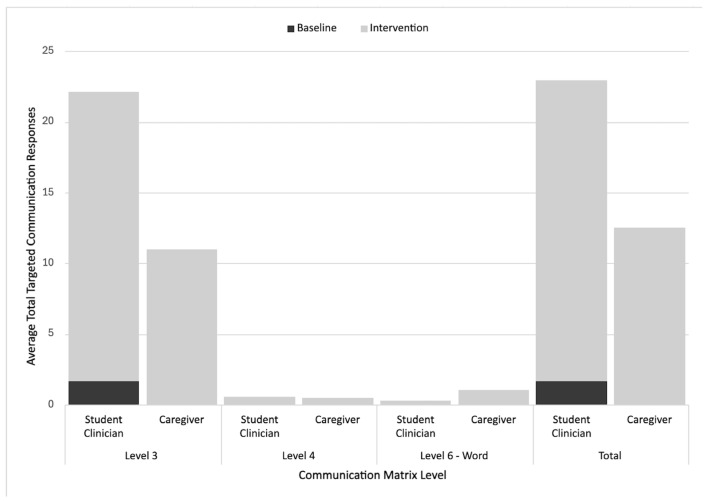
Garrett’s Communication Matrix form response rates (average per 12 min session) across baseline and intervention phases.

**Figure 7:**
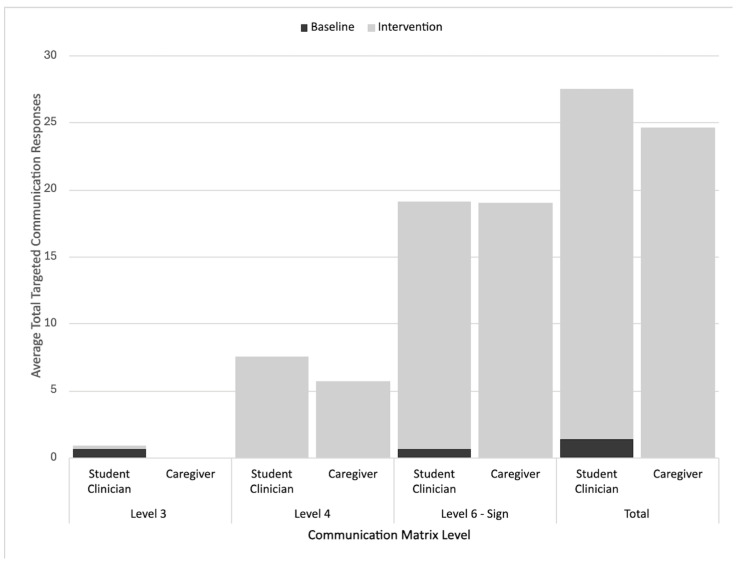
Vince’s Communication Matrix form response rates (average per 12 min session) across baseline and intervention phases.

**Figure 8:**
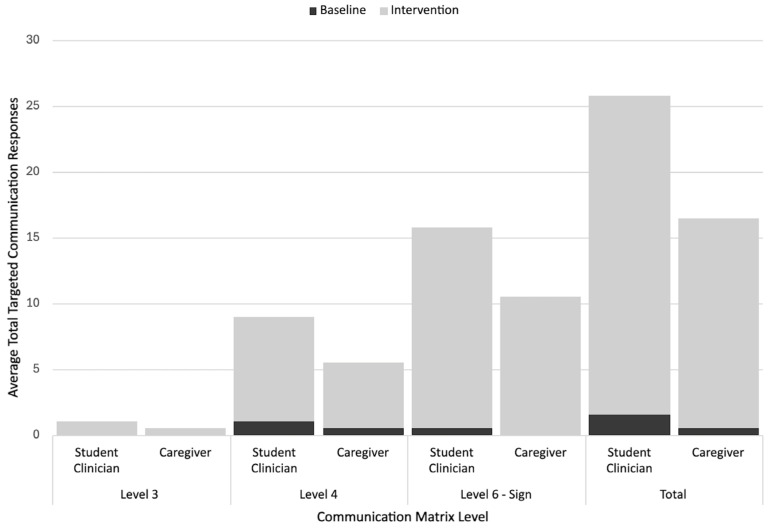
Michelle’s Communication Matrix form response rates (average per 12 min session) across baseline and intervention phases.

**Figure 9:**
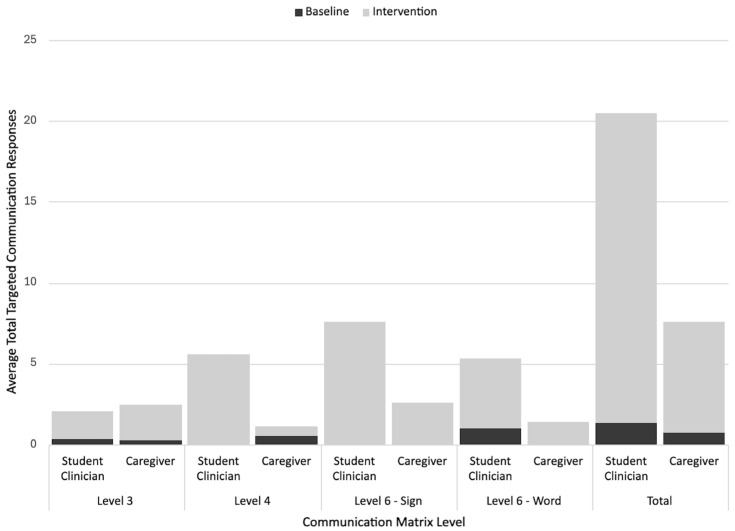
Gustavo’s Communication Matrix form response rates (average per 15 min session) across baseline and intervention phases.

**Figure 10:**
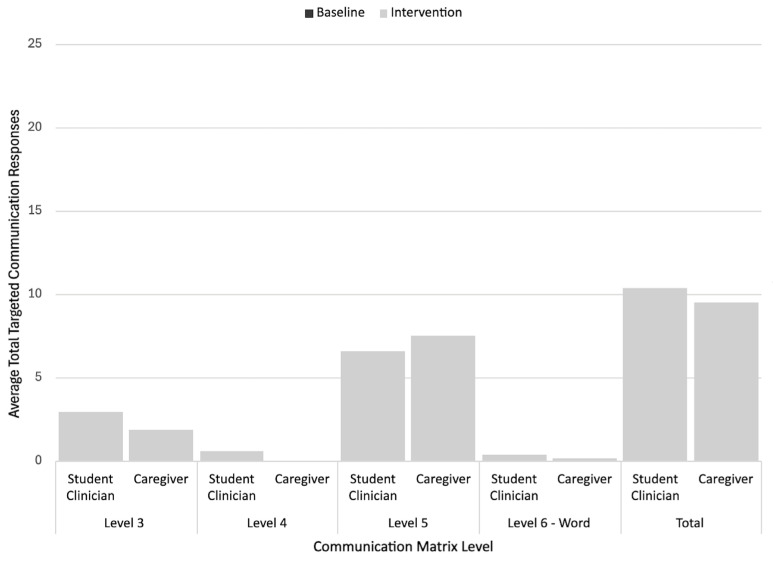
Zandra’s Communication Matrix form response rates (average per 15 min session) across baseline and intervention phases. It should be noted that for Zandra, baseline data for targeted communication responses were zero across all Communication Matrix levels, resulting in no visible baseline bars.

Descriptive analysis of the Communication Matrix results demonstrated that the participants used a variety of response topographies during the intervention. The most targeted communication responses at baseline were unconventional pre-symbolic communication forms (level III). In comparison, four of the five participants (excluding Garret who showed low rates of level VI responses) consistently used symbolic communication forms (levels V–VI) during the intervention, while also continuing to use level III and/or level IV responses. Three of these four participants most often used level VI signs, while Zandra most often used level V aided AAC forms. Additionally, although statistical analyses were not conducted, most participants had higher intervention rates of responding with each targeted Communication Matrix level/context when compared to the baseline. As noted in the graphs featured earlier in this article, the children also had a higher total response rate in the student clinician-led sessions as compared to the caregiver-led sessions. However, there were more variable patterns in terms of the differences between the average rates of specific Communication Matrix level forms used across the different student clinician and caregiver-led contexts. For instance, while Vince had similar average rates of level VI sign responses across the different contexts, Michelle and Gustavo used signs more often with student clinicians, and Gustavo also used more vocal words with his student clinician. Conversely, Zandra had slightly higher average rates of level V (aided AAC) responses with her mother than with her student clinician. The detailed results for each participant are described below. Figure 6, Figure 7, Figure 8, Figure 9 and Figure 10 also display visual comparisons of the Communication Matrix levels across the different phases and contexts.

#### 3.2.1. Garret’s Communication Matrix Level Response Rates 

Garrett’s sessions (both student clinician and caregiver-led) most frequently incorporated level III (unconventional gestures) responses across the baseline and intervention (i.e., 20.42 per student clinician-led intervention session, 11 per caregiver-led intervention session). The level III response rates increased across both contexts from the baseline to the intervention. The rates of level IV (conventional gestures) and VI (vocal words) responses per intervention session were low (1 or less), but increased from 0. Figure 6 further details the Communication Matrix outcomes for Garrett.

#### 3.2.2. Vince’s Communication Matrix Level Response Rates 

Vince’s intervention sessions included primarily level IV (conventional gestures) and VI (signs) responses across the student clinician-led and caregiver-led sessions, with his level VI responses having the highest rates (i.e., 18.4 per student clinician-led intervention session, 19 per caregiver-led intervention session). Level III responses were only elicited by the student clinician during both the baseline and intervention sessions. This rate was still averaged to less than one per session, showing it to be the least frequently used type of communication. Both the student clinician- and caregiver-led sessions showed an increased use of level IV and level VI responses from the baseline to the intervention. Figure 7 further details the Communication Matrix outcomes for Vince.

#### 3.2.3. Michelle’s Communication Matrix Level Response Rates 

The most frequently occurring type of communication response for Michelle was level VI (signs) for both the student clinician (i.e., 15.5 per session) and caregiver-led (i.e., 10.25 per session) intervention sessions, with increases in this response during both contexts from the baseline to the intervention. The other two communication levels tracked (i.e., III, IV) also showed increases in the rate from the baseline to the intervention across different contexts (with low rates of level III responses). Figure 8 further details the Communication Matrix outcomes for Michelle.

#### 3.2.4. Gustavo’s Communication Matrix Level Response Rates 

The most prevalent communication response level for Gustavo during the student clinician- and caregiver-led sessions was level VI responses (signs; i.e., 7.57 per student clinician-led session, 2.6 per caregiver-led session), followed by level VI responses (vocal words). Like with signs, the average rates of vocal word use and level VI conventional gesture use were higher with his student clinician than with his grandmother. Conversely, he had slightly higher rates of level III responses with his grandmother. Level VI forms (signs and words) and level III forms were used more often in intervention sessions than the baseline sessions across different contexts. His level IV responses increased from the baseline for the student clinician sessions and remained similar to the baseline levels during the caregiver-led sessions. Figure 9 further details the Communication Matrix outcomes for Gustavo.

#### 3.2.5. Zandra’s Communication Matrix Level Response Rates 

At baseline across the different contexts Zandra did not use any targeted communication responses. During her intervention sessions, she most frequently used level V concrete communication (aided AAC) targets. This was consistent throughout the student clinician- and caregiver-led sessions. It is noted that at this level, the caregiver-led sessions had higher rates of the target use (i.e., 6.55 per student clinician-led intervention session, 7.5 per caregiver-led intervention session). Conversely, she used level III responses (unconventional gestures and vocalizations) slightly more frequently with the student clinician than with her mother. Level VI responses (vocal words) occurred at very low rates during both the student clinician- and caregiver-led sessions. Figure 10 further details the Communication Matrix outcomes for Zandra.

## 4. Discussion

The positive child communication outcomes reported in this study extend similarly positive adult participant findings from prior NDBI cascading coaching programs (Gallegos et al., 2024; Gevarter et al., 2021; McGuire et al., 2025; Meadan et al., 2020). Specifically, this study demonstrated functional relationships between NDBI cascading coaching and autistic preschoolers’ total targeted communicative responses and unprompted responses across both student clinician and caregiver-led contexts. Very large effect sizes were observed for four of the five participants across the different variables and contexts. Gustavo had very large effect sizes for the total targeted communication responses across the different contexts and large effect sizes for unprompted targeted communication responses across the different contexts. Notably, the data also indicates that for most participants and contexts, the levels of unprompted responding tended to only be slightly below (or, in some instances, equal to) the total response rate.

This study also provides further support for the use of NDBIs in regard to Hispanic autistic children from both English-speaking and Spanish-speaking homes (Gallegos et al., 2024; Gevarter et al., 2021; Lopez, 2024; McGuire et al., 2025; Meadan et al., 2020; Pickard et al., 2024). It should be noted that like other studies involving Spanish-speaking families (e.g., Lopez, 2024; Pickard et al., 2024), extensive cultural and linguistic adaptations for the two child participants (and their caregivers) who came from Spanish-speaking homes were implemented.

Unsurprisingly, the data indicate that the children used targeted communication responses more often in the student clinician-led sessions than with their caregivers, which is aligned with student clinicians’ overall higher rates of NDBI technique use identified in the prior studies (Gallegos et al., 2024; McGuire et al., 2025). This finding also supports prior research suggesting that clinician-mediated interventions have larger effects on autistic children’s communication outcomes than parent-mediated interventions (Sandbank et al., 2020). However, caregiver-mediated sessions still demonstrated large-to-very large effect sizes across both dependent variables. Additional benefits of caregiver-mediated interventions (e.g., generalization of skills across environments, increased caregiver empowerment and knowledge, ability to meet cultural needs) must also be considered (Lopez, 2024; Rieth et al., 2018; Zwaigenbaum et al., 2015). Furthermore, lower levels of responding during caregiver sessions may also reflect the multiple roles caregivers play beyond the role of interventionist (Gallegos et al., 2024). For instance, in some cases, researchers have observed that caregivers appropriately reduced targeted communication temptation use when their child was not engaged in play or was seeking other forms of attention or interaction (e.g., physical affection; Gallegos et al., 2024).

Regarding the secondary research question, this study provides valuable initial data indicating that when student clinicians and caregivers are taught to incorporate neurodiversity-affirming principles surrounding multimodal communication (M. Wang et al., 2023), autistic children respond to communication temptations using a range of response topographies (e.g., gestures, aided AAC, signs, and vocal words). Although most participants tended to have one preferred communication modality, they also used other forms during the intervention. Furthermore, the data indicate that accepting response forms (e.g., unconventional and conventional gestures) viewed as developmentally simpler on the Communication Matrix (Rowland, 2011) did not inhibit the use of response forms considered developmentally more advanced (e.g., aided AAC, signs, vocal words). For all the participants, except Garrett (who used mostly level III responses), symbolic communication forms, including level V concrete symbols (Zandra) or level VI abstract symbols (Vince, Michelle, Gustavo), were the most used forms during the intervention. Notably, Garrett was the youngest participant and he and Zandra were the two participants reported to rely primarily on level III responses at baseline. Gustavo was the only participant who frequently used vocal words during the intervention (both Zandra and Garret used these at very low rates) and was also the participant reported to have the highest emerging vocal word use at baseline.

Interestingly, despite the overall finding that the children used more targeted communication responses with student clinicians compared to caregivers, there were no consistent patterns indicating whether the children were more likely to use developmentally more advanced communication levels in one context over another. For instance, although Gustavo and Michelle used more level VI sign responses with their student clinician than with their caregiver, Vince’s sign use was similar across different contexts. Zandra also used more level VI aided AAC responses with her mother than with her student clinician. These findings do, however, align with the individualized decisions made during the joint planning sessions that reflected the caregiver’s perspectives. For instance, Vince’s mother reported a high level of comfort and experience with introducing new signs to her son. In comparison, Gustavo’s grandmother indicated initial hesitancy regarding prompting signs (which his student clinician had already introduced) vs. accepting his existing level III–VI response forms. Importantly, he still showed increases in the rate of level VI sign and vocal word responses with his grandmother. In Zandra’s case, her mother indicated a strong preference for prompting aided AAC vs. accepting existing level III response forms. In contrast, during qualitative interviews that took place as part of the prior study, Zandra’s student clinician reflected upon the value of being able to accept level III response forms when Zandra appeared frustrated or showed signs of prompt aversion, which was a technique discussed with the lead instructor during coaching (McGuire et al., 2025).

### 4.1. Implications for Practice

The findings from this study demonstrate that autistic children show positive communication outcomes when clinicians and caregivers embed principles aligned with neurodiversity-affirming/strengths-based values within NDBIs. First, although this study inherently provides external evidence on NDBIs, other components of EBPs were incorporated into the study design. For instance, as part of the joint planning stage of coaching, researchers specifically elicited student clinician expertise and caregiver perspectives regarding communication response topographies and prompt types. This led to the use of highly individualized targeted communication strategies for the participants. Although such individualization likely impacted the variability in responding, it is appropriate to target outcomes that have a high likelihood of being elicited and responded to by specific communication partners (Brodhead et al., 2020). The data from this study demonstrate that even with these individualized components, the children showed increased use of unprompted responses and developmentally more advanced communication forms.

Caregiver perspectives were also incorporated via the use of predetermined and individualized cultural adaptations (e.g., coaching provided in Spanish, adding meal routines to Zandra’s sessions). To ensure cultural responsivity in regard to NDBIs, clinicians working with autistic children and their families should incorporate joint planning (including a discussion of multimodal communication response forms) and make additional cultural adaptations when needed (Douglas et al., 2024; Gallegos et al., 2024; Gevarter et al., 2021; M. Wang et al., 2023). Additionally, given the differences in the child outcomes across the caregiver and student clinician-led sessions, clinicians should be prepared to adjust the goals and outcomes for caregiver-mediated interventions and allow for more gradual changes in child communication that correspond to adult learning.

Another practical implication regarding embedding the EBP model within NDBIs involves the incorporation of client perspectives and internal evidence. In this study, child preferences were actively embedded within selected routines and activities. Specifically, the RAISD (Fisher et al., 1996, 2021) was used to individualize the intervention by not only selecting preferred activities and materials, but also considering sensory preferences and aversions within the intervention, which is a strengths-based strategy recommended by autistic individuals, researchers, and related professionals (Donaldson et al., 2017). When satiation with materials was observed, the lead instructor provided student clinicians with feedback regarding ways to vary activities to ensure continued motivation. However, when a motivating operation could not be established, the instructor reminded the student clinicians to follow the child’s lead and consider other targeted temptations. Additionally, the lead instructor used ongoing observations of student clinician sessions to make suggestions based on internal evidence. For example, if the lead instructor observed that a child was not responding to a particular prompt or may need more waiting time, these suggestions were provided in real-time during the coaching sessions.

Naturally, incorporating client and caregiver perspectives also aligned with the use of several neurodiversity-affirming principles (M. Wang et al., 2023). First, this study taught student clinicians and caregivers to elicit and respond to multiple forms of communication, which has been recommended by disability groups and autistic individuals (Donaldson et al., 2017; Gaddy & Crow, 2023; National Joint Committee for the Communication Needs of Persons with Severe Disabilities, 2024). In practice, clinicians can use the Communication Matrix (Rowland, 2011) not only as an initial assessment to inform target selection, but also as an ongoing evaluation tool that can support progress monitoring for multiple forms of communication.

Several additional neurodiversity-affirming principles used in this study can also be incorporated into practice. First, by implementing the principle of client autonomy and agency (Gaddy & Crow, 2023), clinicians can end intervention sessions when assent to participate is withdrawn (which occurred once during a session with Gustavo) and still see overall positive effects of the intervention. Additionally, clinicians using NDBIs should select communication temptations that align with participants’ functional communication, self-advocacy, and participation needs during play routines (e.g., asking for assistance in order access or use a preferred item, rejecting non-preferred items, making it clear when they want a routine to continue). Furthermore, clinicians must consider when it is and is not appropriate to prompt communication. For instance, in this study, during group instructions with student clinicians, the lead instructor discussed instances in which using more restrictive prompts (e.g., physically graduated guidance) would be inappropriate, such as when the child shows aversion or withdraws assent. Hand-under-hand vs. hand-over-hand models were also discussed. Zandra’s observed and reported physical prompt aversion for signs and gestures (and reported aversion to her mother prompting vocal speech) also informed the decision to introduce aided AAC during the intervention.

During coaching, the lead instructor also pointed out situations in which a child showed a prompt aversion to the prompt or did not appear happy, relaxed, and engaged (HRE; Gover et al., 2022; Hanley, 2021). Ensuring that a child is in a HRE state has been suggested as a prerequisite for learning that also enables children to feel safe and in control (Hanley, 2021). In this study, Zandra often used higher pitched persistent vocalizations or more forceful unconventional gestures paired with grimacing when she was not in a HRE state. During such times, the coach encouraged the student clinician to accept these unconventional responses rather than attempt to prompt aided AAC. This approach aligns with suggestions regarding how to help children return to HRE states (Hanley, 2021). Alternatively, Zandra’s mother continued to prompt AAC even at times when Zandra did not always show signs of being in a HRE state (as evidenced by Zandra’s lower rates of level III responses and higher rates of level V responses with her mother). In practice, clinicians may need to provide explicit instructions and real-time feedback to caregivers regarding recognizing signs of prompt aversion or when a child is not in a HRE state. In these contexts, caregivers may need to be taught to gradually shape more conventional communication forms by reinforcing lower-level response forms when HRE is not demonstrated and prompting more advanced forms when the child is regulated. Other caregivers, like Gustavo’s grandmother (with whom Gustavo used more lower-level response forms), may benefit from more focused instruction on prompting higher-level communication forms when the child is showing signs of being in a HRE state.

### 4.2. Limitations and Future Research Directions

This study had several limitations that suggest important future research directions.

First, regarding What Works Clearinghouse (WWC, 2022) standards for multiple baseline/probe designs, this study would only meet the required standards with reservations. Although three tiers within a multiple baseline design might be the simplest method for demonstrating effects across at least three distinct points in time (WWC, 2022), this may not always be the ideal approach when considering implementation factors (Lanovaz & Turgeon, 2020). In this study, although there were only two tiers in each implementation of the design, because it was replicated five times, the design meets the standards by demonstrating 10 demonstrations of the effect replicated across distinct points in time. However, because not all of the phases included five data points, the design would only meet the required standards with reservations. It has been noted that requiring five data points may pose practical difficulties that hinder the feasibility of conducting research in authentic, real-world settings (Harris et al., 2019). In this instance, the limited timeframe of the summer clinic (impacted by constraints tied to the program’s funding and student clinician availability) restricted the duration available for baseline data collection and made it impractical to implement cascading coaching through a third tier for each participant group.

Additional limitations also stem from the brief nature of the intervention. For example, due to time constraints, decisions regarding when to begin the intervention in caregiver-led contexts were based on increases in student clinicians’ use of NDBI techniques rather than child outcome measures. No data relating to maintenance or generalization to home/other preferred materials were collected. Time constraints also prevented teaching the adult participants to use more advanced NDBI skills (e.g., shaping, prompt fading) that could impact child outcomes. For instance, the children may have demonstrated greater increases in the use of independent responding or more advanced forms of communication. Time constraints also informed the decision not to introduce entirely new forms of communication during the intervention for most participants, except Zandra. As noted in the prior study, although introducing the aided AAC device after the baseline session presented a design limitation, the decision was made to allow for ethical, neurodiverse-affirming, and culturally responsive treatment (McGuire et al., 2025). Earlier introduction of these forms across participants should be considered in future cascading coaching programs. Additionally, researchers should replicate this study in home and community-based settings (using additional play materials to enhance generalization) across greater periods of time and using more advanced NDBI methods. The introduction of SGD-based responses may be particularly beneficial given their higher likelihood of reinforcement across communication partners as compared to sign responses (Brodhead et al., 2020). In addition to examining maintenance over time, a longer study length might also allow for further exploration of additional social validity measures. Importantly, social validity findings from both prior studies indicated that all the caregivers and student clinicians described positive treatment outcomes for the child participants (Gallegos et al., 2024; McGuire et al., 2025). Following up with caregivers regarding the long-term outcomes for children could provide additional valuable information. Additionally, autistic adults could be asked to rate the social validity of the interventions in terms of its alignment with neurodiverse-affirming care.

Limitations also exist within the study population as this study included only five children, all of whom were Hispanic preschoolers, who were either minimally verbal or non-speaking. Additional research should include children from other underrepresented groups (e.g., Native Americans) and aim to extend the findings to children with more varied language abilities. For example, replicating the study with echolalic children would help fill research gaps in terms of strengths-based approaches to echolalia (Blackburn et al., 2023; Stiegler, 2015). Furthermore, future research should explore long-term child communication outcomes and other dependent variables. For example, this study did not examine how often children using non-speech forms paired these topographies with vocal approximations or non-word vocalizations. Although this data could not be reliably analyzed due to some participants using low-volume vocalizations that were difficult to discern on the video recordings, anecdotally the researchers observed all the participants pairing non-word vocalizations with signs or gestures on occasion. Zandra was also observed to sometimes imitate digitized speech output that occurred when she made an AAC response, but, in these instances, researchers only coded the AAC response (vocal words were coded only when this was the initial response). Similarly, although Gustavo was anecdotally observed to imitate full-word speech models provided by his student clinician or grandmother after they reinforced signs or gestures, the data involving these post-response imitations were not formally collected or analyzed. It is also important to note that this study only analyzed specific communication targets that occurred in response to adult participants’ targeted communication temptations. Tracking all communication forms used across the sessions would provide additional information. Furthermore, data on the children’s HRE states across the sessions was not specifically collected. Whole interval recording could be used to track such data. Inclusion of HRE data would provide more information on the social validity of the intervention and provide evidence that NDBIs can support human dignity, quality of life, and well-being (Schuck et al., 2022).

The small sample size in this study and the nature of the data collected also prevented the use of parametric or non-parametric statistical analyses of Communication Matrix (Rowland, 2011) data and may limit its generality across a wider range of autistic children with varying linguistic profiles. Researchers should replicate the use of the Communication Matrix as a descriptive analysis tool for tracking multimodal communication topographies across baseline and intervention sessions. However, the use of larger sample sizes or alternative research designs would also allow for the exploration of how the Communication Matrix could be used to evaluate significant changes in specific topographies or overall response variability. Regardless, to better align with neurodiversity-affirming approaches, researchers should incorporate multimodal communication considerations into intervention procedures and data collection processes (Gaddy & Crow, 2023; M. Wang et al., 2023). NDBI researchers should also expand upon the additional neurodiversity-affirming principles used in this study (e.g., honoring assent withdrawal) in future studies (M. Wang et al., 2023). Social validation measures that evaluate how well these principles are incorporated into NDBI research (developed alongside autistic individuals and their families) should also be included.

Finally, it is important to note that in this study, some instructions and feedback that the student clinicians received from the lead instructor were not explicitly defined in the caregiver coaching protocols and fidelity checks. For instance, although the student clinicians were required to discuss prompt selection with the caregivers during the joint planning process, a discussion of when to use lesser or more restrictive prompts (which the lead instructor described to the students) was not an explicit requirement. Similarly, although the student clinicians were required to provide positive and constructive feedback on the caregivers’ use of NDBI techniques (both during and after the play observation), providing feedback on when to accept lower-level response forms vs. when to prompt more advanced forms was also not an explicit requirement. Further analysis of the content and quality of the lead instructor’s feedback vs. student clinician’s feedback would provide additional information regarding whether differences in feedback impact child outcomes in cascading coaching programs. To ensure that neurodiversity-affirming principles are embedded across different contexts, explicit caregiver coaching surrounding HRE and prompt hierarchies/aversions should also be included in protocols and fidelity checks.

## 5. Conclusions

This study provides preliminary evidence that culturally adapted NDBIs, delivered by both student clinicians and caregivers, can have positive effects on the communication outcomes of minimally verbal Hispanic autistic preschoolers. By embedding neurodiversity-affirming and culturally responsive practices, the intervention supported increases in the children’s use of total and unprompted communication responses across a range of topographies (i.e., gestures, signs, aided AAC, and natural speech). Importantly, accepting less advanced forms of communication did not inhibit the use of more symbolic modalities. The originality of this research lies in its focus on child-level outcomes within culturally adapted cascading coaching models, its inclusion of both English- and Spanish-speaking families, and its use of the Communication Matrix to track diverse communication forms. The findings underscore the importance of individualized, family-centered approaches and highlight the potential of brief interventions to yield meaningful gains. Given the study limitations, future research should expand this work to broader populations, explore long-term outcomes, and further integrate neurodiversity-affirming principles, such as honoring child autonomy and supporting HRE states in early autism interventions. By doing so, we can move toward a more inclusive, ethical, and effective practices that honor the voices and needs of autistic children and their families.

## Figures and Tables

**Table 1:** Child participant characteristics.

Child	Age	Ethnicity, Gender, and Primary Home Languages	Diagnostic Status	CARS-2 or GARS-3 Score	VABS-III Age Equivalents	Communication Matrix Forms Reported Prior to the Study
Garrett (Study 1)	2;4	Hispanic male; English	ASD diagnosis	43.5 (severe ASD symptoms)	Receptive: 0;9 Expressive: 0;8	Level III (unconventional communication) Emerging Level IV (conventional gestures) Some emerging Level VI (abstract symbols–words)
Vince (Study 1)	2;6	Hispanic male; English	On waiting list at time of study; ASD diagnosis after study	32.5 (mild-to-moderate ASD symptoms)	Receptive: 1;4 Expressive: 0;0	Level IV (conventional gestures) and Level VI (abstract symbols–signs)
Michelle (Study 1)	2;8	Hispanic female; English	On waiting list at time of study; ASD diagnosis after study	34.5 (mild-to-moderate ASD symptoms)	Receptive: 1;5 Expressive: 1;3	Level IV (conventional gestures) and Level VI (abstract symbols–signs)
Gustavo (Study 2)	2;11	Hispanic male; Spanish and English	Independent ASD diagnosis	34.5 on CARS-2 (mild-to-moderate ASD)	Receptive: 1;0 Expressive: 0;11	Level III (unconventional gestures) and Level IV (conventional gestures) with emerging Level VI (signs and words)
Zandra (Study 2)	4;3	Hispanic female; Spanish	Independent ASD diagnosis	94 on GARS-3 (Level 2 requiring substantial support)	Expressive: 1;0 Receptive: 0;6	Level III (unconventional communication)

**Table 2:** Targeted temptations + communicative response forms across different Communication Matrix levels.

	Targeted Temptations	Level III Responses	Level IV Responses	Level V Responses	Level VI Signs	Level VI Vocal Words
Garrett (Study 1)	Give choices; items requiring assistance; inadequate portions; interrupting routines	Hand item to adult for help; reach	Pointing	n/a	MORE	*Go*
Vince (Study 1)	Giving choices; items requiring assistance; hiding or concealing items; taking a turn	n/a	Pointing	n/a	HELP, CAR, MY TURN, OFF, OPEN	
Michelle (Study 1)	Giving choices; inadequate portions; offering non-preferred items	n/a	Pointing, head shake no, head shake yes	n/a	PUZZLE, MORE, WATER, OPEN, BLOCK	
Gustavo (Study 2)	Interrupting routines + carrier phrase (1, 2….); items that require assistance; hiding or concealing items; giving choices	Push item away, touch item, reach	Point	n/a	TRES (fingers up to mean THREE); MAS (MORE) AYUDA (HELP)	*Uno* (*one*) *Tres* (*three*) *Ayuda* (*help*)
Zandra (Study 2)	Inadequate portions; interrupting routines; items that require assistance; hiding or concealing items	Persistent vocalizations (e.g., *ah-ah-ah*); push away items; hand items to adults; guide adults’ hands		Variety of single button responses with Proloquo2Go (e.g., Spanish words for gummy bear, music toys, book, water, etc.)		*Taza* (*cup*), *Agua* (*water*)

## Data Availability

Original data tables for the single-case experimental design are available upon request from the corresponding author.

## Notes

1 The authors are intentionally switching between identity first language and person first language to acknowledge the diverse perspectives on language preferences.

2 While the terms *Hispanic*, *Latino*, and *Latina* are used in this work to reflect the self-identification of the adult participants, the author acknowledges the evolving and complex nature of racial, ethnic, and cultural terminology, and recognizes that alternative terms, such as *Latinx*, *Latine*, and *Latin@,* are also used in various contexts.

3 While non-verbal is often used in the literature, we will intentionally also use the term non-speaking as this is often preferred in disability communities.
